# Xenotransfusion with packed bovine red blood cells to a wildebeest calf (*Connochaetes taurinus*)

**DOI:** 10.4102/jsava.v89i0.1669

**Published:** 2018-10-17

**Authors:** Roxanne K. Buck, George F. Stegmann, Luke A. Poore, Tahiyya Shaik, Travis Gray, Gareth E. Zeiler

**Affiliations:** 1 Department of Companion Animal Clinical Studies, University of Pretoria, South Africa

## Abstract

A 4-month-old female blue wildebeest (*Connochaetes taurinus*) was presented for bilateral pelvic limb fracture repair. Clinical examination under anaesthesia revealed a water-hammer pulse and a haematocrit of 0.13. A xenotransfusion was performed using bovine (*Bos taurus*) erythrocytes because of inability to acquire a wildebeest donor. Clinical parameters improved following transfusion and the post-operative haematocrit value was 0.31. The wildebeest remained physiologically stable with a gradually declining haematocrit for the next three days. On the third post-operative day, the wildebeest refractured its femur and was humanely euthanised because of the poor prognosis for further fracture repair. Xenotransfusion using blood from domestic ruminants represents a life-saving short-term emergency treatment of anaemic hypoxia in wild ungulates. Domestic goats could be used as blood donors for rare ungulates where allodonors are not available.

## Introduction

Oxygen is required for efficient production of energy in the form of adenosine triphosphate (ATP), which is required to drive many cellular functions (Ward 2006). When oxygen supply is insufficient to meet cellular demands, or cells are unable to utilise oxygen, hypoxia and potential tissue damage result. Oxygen delivery (DO_2_) is determined by the product of cardiac output (CO) and arterial oxygen content (CaO_2_). Cardiac output is in turn the product of heart rate and stroke volume. The CaO_2_ is dependent on both oxygen bound to haemoglobin and oxygen dissolved in the blood, as defined by the following equation: CaO_2_ = (1.34 × Hb × SaO_2_) + (0.003 × PaO_2_), where Hb is the haemoglobin concentration, SaO_2_ is the percentage of oxygen saturation and PaO_2_ is the arterial oxygen tension measured in mmHg (Dugdale 2010). Thus, it becomes clear that adequate oxygen delivery depends on both a functioning cardiovascular system and an adequate concentration and saturation of haemoglobin in the blood. To optimise the saturation of the haemoglobin, the body requires functional integration of the cardiovascular and pulmonary systems to allow effective gas exchange at alveolar-capillary junctions.

Inadequate haemoglobin and subsequent anaemic hypoxia can cause mortality in ungulates (Poore et al. 2017). Treatment of anaemia can be achieved with blood transfusion, but this is often discounted in wild ruminants because of perceived difficulties and unfamiliarity. In addition, allodonors are often not available for rare ungulate and wild animal species. Xenotransfusion is the administration of blood products from a different species, performed to provide short-term supplementation of haemoglobin to allow an animal to survive an immediate hypoxic crisis. This case reports the successful use of a xenotransfusion in a wild ungulate and highlights the potential benefits of blood transfusion in wild animals.

## Ethical consideration

This is a retrospective case report detailing the case management and outcome of a client-owned patient that presented at the Onderstepoort Veterinary Academic Hospital (OVAH), based on clinical records. The patient was treated and housed according to standard hospital procedures at all times. Standard client consent was obtained for data to be used for publication.

## Case presentation

**FIGURE 1:**
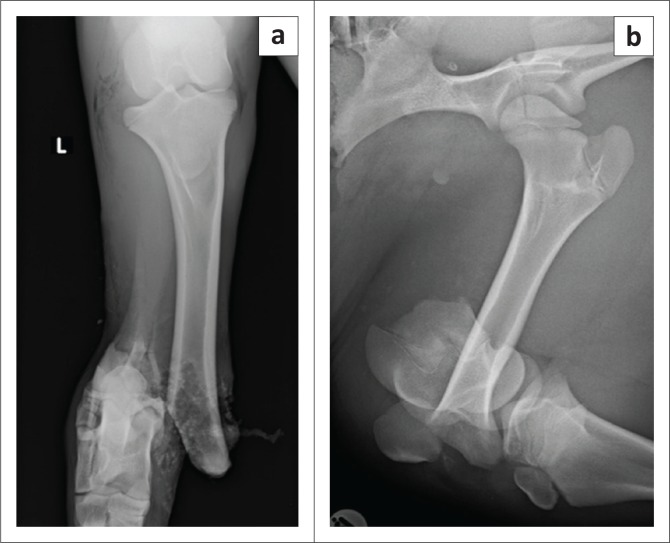
Radiographs of the pelvic limbs of a 4-month-old wildebeest (*Connochaetes taurinus*) showing complete fractures to the (a) left tibia and (b) right femur sustained in a motor vehicle accident.

A fractious 4-month-old female wildebeest (*Connochaetes taurinus*) weighing 70 kg was presented for surgical fixation of bilateral pelvic limb fractures resulting from a motor vehicle accident (Figure 1). Anaesthesia was induced with medetomidine (0.01 mg/kg; 40 mg/mL, Kyron Laboratories, South Africa) and ketamine (5 mg/kg; 100 mg/mL, Ketamine Fresenius, Fresenius Kabi, Midrand, South Africa) intramuscularly. The wildebeest was intubated using a 10 millimetre internal diameter PVC endotracheal tube and anaesthesia was maintained with isoflurane (Isofor, Safeline, Weltevreden Park, South Africa) in oxygen (end-tidal concentration 0.8% – 1.1%). Intravenous fluid support was provided in the form of an isotonic crystalloid (lactated Ringers solution, 10 mL/kg/h) and a hydroxyethylated starch colloid (two 10 mL/kg boluses in the first 3 hours of anaesthesia; Voluven; Fresenius Kabi, Midrand). Clinical examination under general anaesthesia revealed a water-hammer pulse, cold extremities and marked pallor. Clinical parameters and arterial blood gas analysis results are shown in Table 1. Direct arterial blood pressure was satisfactory 2 h after induction of anaesthesia but decreased thereafter to levels associated with hypoperfusion in mammalian species (mean arterial blood pressure less than 60 mmHg [Dugdale 2010]) (Table 1). Arterial blood gas analysis 2 h post-induction revealed moderate acidaemia, with hypercapnoea, hyperlactataemia, mild hypocalcaemia and moderate hyperkalaemia. Intermittent positive pressure ventilation was initiated using flow-controlled ventilation, with a peak inspiratory pressure of 10 cmH_2_O, inspiratory to expiratory ratio of 1:2, tidal volume of 500 mL and initial frequency of 20 breaths/minute (SurgiVet Large Animal Ventilator, Smiths Medical, Dublin, OH, United States). The hypercapnoea was successfully corrected and thereafter the respiratory frequency decreased. Calcium borogluconate (10 mL, 40% m/v, Lionel’s Veterinary Supplies, Johannesburg, South Africa) was administered to treat the hypocalcaemia. Dextrose (1 mL/kg 50% solution) was administered to treat the hyperkalaemia. Analgesia was provided in the form of morphine (0.05 mg/kg IV q2h), meloxicam (0.5 mg/kg SC q48h) and a single bolus of ketamine (1 mg/kg IV 3 h after induction, at the start of surgery). The intra-operative haematocrit was 0.13 (2 h after induction); this decreased to 0.12 (3 h post-induction).

**TABLE 1:** Physiological and blood gas parameters for an anaesthetised blue wildebeest (*Connochaetes taurinus*) calf undergoing isoflurane-in-oxygen anaesthesia for repair of bilateral pelvic limb fractures.

Parameter	Time					
	13:00	14:00	15:00	16:30	21:30^a^	00:00^b^
Heart rate (bepm)	50	50	40	45	40	85
SAP (mmHg)	100	75	95	90	110	-
MAP (mmHg)	70	60	60	65	85	-
DAP (mmHg)	50	35	45	45	70	-
Respiratory rate (brpm)^C^	8	20^c^	15^c^	15^c^	15^c^	40
Temperature (^o^C)	34.6	34.4	34.4	34.8	33.8	35.7
Haematocrit (L/L)	0.13	0.12	0.13	0.13	0.30	0.31
FiO_2_	1.00	1.00	1.00	1.00	0.21	0.21
pH	7.121	7.457	7.593	7.565	7.435	-
PaO_2_ (mmHg)	202.6	225.9	347.5	371.0	75.6	-
PaCO_2_ (mmHg)	110.4	44.7	36.6	37.3	43.2	-
HCO_3_^-^ (mmol/L)	35.2	30.9	30.5	33.0	28.4	-
Base excess (mmol/L)	5.9	6.4	7.2	9.9	3.7	-
Na^+^ (mmol/L)	128.0	124.4	132.0	137.9	136.0	-
K^+^ (mmol/L)	6.7	7.1	4.4	3.2	3.3	-
Ca^2+^ (mmol/L)	1.16	1.03	1.13	1.10	1.13	-
Cl^-^ (mmol/L)	91	91	95	100	101	-
Lactate (mmol/L)	5.8	-	5.2	-	3.3	2.2

Note: Anaesthesia started at 11:00 and continued until 21:30.

SAP, systemic arterial blood pressure; MAP, mean arterial blood pressure; DAP, diastolic arterial blood pressure; FiO_2_, inspired oxygen fraction; PaO_2_, arterial oxygen tension; PaCO_2_, arterial carbon dioxide tension; HCO_3-_, arterial bicarbonate concentration; Na+, arterial sodium ion concentration; K+, arterial potassium ion concentration; Ca_2_+, arterial calcium ion concentration, Cl-, arterial chloride ion concentration; bepm, beats per minute; brpm, breaths per minute.

a , Blood transfusion.

b , Atipamezole administered.

c , Indicates intermittent positive pressure ventilation.

## Management and outcome

A blood transfusion was deemed essential to treat hypoxia, but because of inability to acquire a wildebeest blood donor, a xenotransfusion with bovine blood was performed. Fresh whole blood was collected from a healthy donor cow in citrate-phosphate-dextrose commercial blood collection bags (JMS Medical, Singapore). Because of a minor cross-match (agglutination) reaction, prednisolone (1 mg/kg IM, Bayer, Johannesburg) was administered prior to transfusion and the blood was separated and only packed red cells were administered. Blood transfusion was started 7 h after induction. The blood was administered over 1 h and no reaction to transfusion was observed.

During the transfusion, hypocalcaemia developed. This was detected by apparent prolongation of the ST segment on the electrocardiogram (ECG) with a decrease in heart rate and hypotension. Calcium borogluconate (10 mL) was administered intravenously (IV) and the ECG abnormalities resolved and the heart rate improved.

Marked improvement in mucous membrane colour and pulse quality was noted following transfusion. Diastolic arterial blood pressure improved from 45 mmHg pre-transfusion to 70 mmHg post-transfusion. At the end of the anaesthesia, blood pH was appropriate, haematocrit was 0.31 (3 h post-transfusion), pulse quality was good and mucous membranes were pink and moist. Serum lactate also decreased from 5.2 mmol/L prior to transfusion to 3.3 mmol/L post-transfusion.

Total anaesthesia time was 10.5 h and surgical time was 6.5 h. The wildebeest was hypothermic following surgery and recovery from anaesthesia was prolonged. Atipamezole (2 mg IM, Antisedan, Zoetis, South Africa) was used to antagonise the medetomidine 1 h after the end of the anaesthesia. The wildebeest could hold her head up 1 h post-administration of atipamezole and 2 h after the end of anaesthetic maintenance.

**FIGURE 2:**
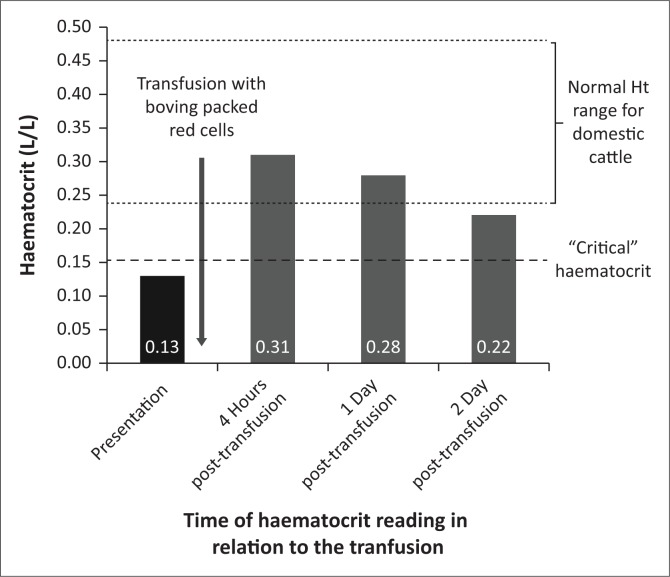
Pictorial representation of haematocrit (L/L) of a 4-month-old wildebeest (*Connochaetes taurinus*) that presented for fracture repair following trauma before and after xenotransfusion.

The wildebeest was kept in a small, padded stall following recovery and observed. Analgesia was provided in the form of morphine (0.05 mg/kg IV q4h) and meloxicam (0.5 mg/kg SC q48h). The haematocrit decreased to 0.28 after 24 h of transfusion and to 0.22 after 48 h (Figure 2). On post-operative day 3, the wildebeest was bright, alert and eating well. She was also physiologically stable (heart rate 90 beats/minute, good-quality peripheral pulses, regular respiration with a frequency of 14 breaths/min, temperature 38 °C) and began attempting to bear weight on both hind limbs. However, she refractured her femur while attempting to walk and the decision was made to humanely euthanise her because of poor prognosis for fracture repair.

## Discussion

Satisfactory oxygen delivery to the tissues relies on both an adequate cardiac output and sufficient arterial oxygen content. Under normal conditions, oxygen delivery far exceeds the oxygen demand of the tissues. This spare capacity allows the body to cope with a fall in DO_2_ without initially compromising aerobic respiration (Nathan & Singer 1999). However, anaerobic respiration will predominate when DO_2_ falls below a critical point or when oxygen demands increase beyond what can be met by oxygen supply and extraction (Nathan & Singer 1999).

The decision to transfuse an anaemic patient is related to the ability of the animal to compensate for the lowered oxygen-carrying capacity of their blood and their oxygen requirements. Generally, with slow developing or chronic conditions, the haematocrit must be exceptionally low (< 0.10) before any clinical signs are seen, as the animal has time to compensate (Divers 2005). In an acute setting, however, there is no time for adaptation. Although domestic ruminants have been shown to tolerate acute blood loss exceptionally well (Balcomb & Foster 2014), a haematocrit of less than 0.15 leads to compromised myocardial oxygenation and a haematocrit below 0.12 is considered ‘critical’ (Divers 2005). Myocardial oxygen demands far exceed those of any other tissue. While the normal oxygen extraction ratio of tissues at rest is 20% – 30%, that of the myocardium is 70% – 80% (Ramanathan & Skinner 2005). This makes the myocardial tissue particularly sensitive to decreases in coronary artery DO_2_. Interestingly, the mean haematocrit of headshot wildebeest was shown to be 0.43 ± 0.071 (Drevemo, Grootenhuis & Karstad 1974). This is considerably higher than domestic cattle (where the mean for Zebu cattle is 0.30 ± 0.049) (Turkson & Ganyo 2015), which may suggest that wildebeest are actually less able to tolerate a low haematocrit than some other ungulate species, although the critical haematocrit in this species has not been investigated.

In addition to blood tests, the decision to transfuse should be guided by clinical assessment. Clinical signs such as weakness, pallor, tachycardia and respiratory distress are suggestive of shock and should prompt transfusion in anaemic animals (Ermilio & Smith 2011). Lactate accumulation is an indicator of anaerobic metabolism and can be used clinically as a quantifier of oxygen debt (Rixen & Siegel 2005).

In this case, the primary cause of anaemia was likely acute haemorrhage secondary to trauma. Blood loss may have occurred from the open fracture to the left tibia. In addition, mass haemorrhage was evident in the muscle fascial planes and subcutis surrounding the fracture to the right femur. However, a number of other factors may have contributed. The young age of the animal may have resulted in a lower haematocrit value prior to injury. In dogs, it has been documented that haematocrit drops after birth to below adult levels as a result of foetal erythrocyte destruction in combination with rapid growth (Lee, Brown & Hultzer 1976). However, this was not demonstrated in calves, where the haematocrit remained within the normal range of adult cattle for the first 6 months of life (Brun-Hansen, Kampen & Lund 2006). Additionally, fracture of bones with a substantial medullary cavity (tibia and femur) may have decreased erythrocyte stores available for recruitment. Although not obvious in this case, poor nutrition or disease could also play a role. No blood-borne parasites were detected on blood smear examination and the wildebeest was in an acceptable body condition. However, the wildebeest may have had pre-existing anaemia or weakness prior to being hit by the car and these factors may have been the reason why she could not avoid the car.

The decision to perform a transfusion in this case was based on the pallor, water-hammer pulses and the limited improvement in lactate levels following intravenous fluid therapy. These factors were all suggestive of an oxygen supply that could not meet requirements. Tachycardia was not seen, and the heart rate remained 50 beats/min for the first 4 h of anaesthesia, likely as a result of medetomidine being administered as an immobilisation agent (Kastner 2006). This perception was supported by the increase in heart rate seen following atipamezole administration. A further factor was that blood loss occurred acutely, thus allowing minimal time for acclimatisation. An additional consideration was the low diastolic blood pressure, as the coronary perfusion pressure is dependent on the gradient between diastolic aortic pressure (DAP) and right atrial end diastolic pressure. Low diastolic pressure decreases the gradient, thereby compromising myocardial perfusion. This is particularly dangerous in low oxygen states given the supply-dependent nature of myocardial oxygenation (Ramanathan & Skinner 2005). A combination of decreased DAP and anaemia combined with clinical indicators of shock (pallor, weak pulses and cold extremities) thus hastened the decision to perform a transfusion. Throughout anaesthetic maintenance, the wildebeest was on 100% oxygen, which would increase the PaO_2_. However, given the exceptionally small contribution of PaO_2_ to CaO_2_ (3%), without increasing the erythrocyte number, and thereby haemoglobin concentration, the CaO_2_ would not be increased sufficiently by oxygen support alone. Given the correction of the hyercapnoea and the normal bicarbonate and chloride levels, it could be assumed that haemoglobin was primarily acting in oxygen transport and not as a buffer and there was no impediment of oxygen diffusion at tissue level (Dugdale 2010).

An important consideration when interpreting haematocrit values is the effect of anaesthesia. Both general anaesthesia and sedation have been shown to cause a precipitous drop in haematocrit as a result of drug action and a change in fluid distribution. Splanchnic vasodilation results in sequestration of erythrocytes in the spleen, lungs and other organs (Wilson et al. 2004). In addition, vasodilation increases vascular compliance and thus decreases extravasation of fluids to the interstitium. This combines with a decrease in cardiac output, which in turn decreases glomerular filtration, leading to relative volume overload (Lahsaee, Ghaffaripour & Hejr 2013). Hormone-mediated effects, particularly suppression of catecholamine and cortisol release, also contribute through blood volume redistribution (Zlateva & Aminkov 2014). These factors, in combination with surgical blood loss and erythrocyte dilution by intravenous fluids, need to be considered in the intra-operative period. Thus, a low initial haematocrit can quickly decrease even further. This is particularly important in wild ungulates where sedation or anaesthesia is required before a clinical examination can be performed.

Usually, general anaesthesia decreases DO_2_ through the decrease in haematocrit and a decrease in cardiac output. However, this is usually accompanied by a corresponding decrease in oxygen demand because of drug-induced lowered metabolism (Dugdale 2010). It should be kept in mind, however, that the oxygen requirements of the myocardium and skeletal muscle are also far greater during exercise than at rest (Ward 2006). This is important to remember with wild animals where excitement at capture (darting) can increase oxygen demands. Pain and anxiety have also been shown to increase oxygen consumption in humans through restlessness and increases in circulating catecholamines (Ward 2006).

While allotransfusions are the current norm, the administration of blood from a different species is not a novel concept. The first documented blood transfusion to a human was performed by Jean-Baptiste Denis in 1667 using blood from a domestic sheep and it was not until 150 years later that the first allotransfusion was performed in humans (Roux, Sai & Deschamps 2007).

Xenotransufsion of cats with dog blood was extensively studied in the 1960s and has provided much of the modern knowledge of the practice (Bovens & Gruffydd-Jones 2012). No severe acute adverse reactions were seen following a single transfusion (Clark & Kiesel 1963) and the transfusion of cats with dog blood is still an accepted emergency practice (Bovens & Gruffydd-Jones 2012). However, it should be noted that in cats given dog blood, the lifespan of xenotransfused erythrocytes is significantly shorter than that of allo-erythrocytes (4 days for dogs erythrocytes versus 30 days for cat erythrocytes) (Lautieet al. 1969). Domestic dog blood has been transfused successfully to wild dogs *Lycaon pictus* (van Heerden et al. 1994).

Whole blood transfusion is relatively rare in ruminants, largely because of the cost-restrictive environment encountered with farm animal practice (Constable 2003). It has been noted that the survival of transfused erythrocytes in ruminants is shorter than in dogs and horses as allo-antibodies are produced within four days, resulting in removal of transfused erythrocytes from circulation (Schnappauf, Di Giacomo & Cronkite 1965). This illustrates the short-term benefit of transfusion. However, normal bone marrow regeneration is generally effective within five days (Smith 2009). A transfusion may thus provide a useful short-term life-saving increase in the oxygen carrying capacity of blood, which may able to bridge a gap caused by acute blood loss. Unfortunately, a reticulocyte count was not performed in this case as this may have provided an indication of the wildebeest’s own haemopoietic response.

Domestic ruminants are good potential donors for wild ungulates, as they are readily available and blood collection and transfusion can be performed in field settings. A grey duiker has even been successfully given goats blood (J. van Heerden pers. comm., July 2018). Generally, a large volume, up to 10 mL/kg – 15 mL/kg may be drawn from a donor (Divers 2005). Aseptic technique should always be maintained during blood collection and blood administration should always be performed through a blood filter set to remove microemboli (Ermilio & Smith 2011). The donor should be healthy and blood should always be examined for blood-borne parasites prior to administration (Ermilio & Smith 2011).

Haemolytic transfusion reactions remain a possibility and xenotransfusion is complicated by antigenic differences across species. Cross-matching should be done where possible. Complement activation methods are preferred because of low concentrations of circulating antibodies rendering agglutination methods of cross-matching less useful (Balcomb & Foster 2014). It has been suggested that goat blood may be less antigenic than sheep or cattle blood because of the absence of J- (cattle) and R-factors (sheep), which are likely to cause reaction even in naïve recipients (Smith 1991). The choice of cattle blood in this case was based on availability of a donor animal, although it may have been prudent to choose goat blood, as there was a minor cross-match reaction (donor plasma reacting to the recipient erythrocytes). In an attempt to prevent a haemolytic reaction, the fresh whole blood was spun down and only packed red blood cells were administered to reduce the number of antibodies supplied by removing the plasma. In addition, the wildebeest was treated with prednisolone to decrease immune responses prior to transfusion. Despite this minor cross-match reaction, transfusion reactions in ruminants are exceedingly rare because of low circulating quantities of iso-antibodies, and transfusion can usually be performed safely in naïve animals (Balcomb & Foster 2014).

Hypocalcaemia is a common complication when using citrate-based anticoagulants because of calcium binding. Treatment with calcium borogluconate is usually curative (Balcomb & Foster 2014). Additional complications of transfusion could include parasite or disease transmission, infection, allergic reactions, febrile reactions and transfusion-related acute lung injury (Chohan & Davidow 2015).

The xenotransfusion was successful in raising the haematocrit, although a gradual decline in haematocrit was seen over the following 48 h. Part of the reason for this is redistribution, which occurs following transfusion, with a shift of fluid from the intracellular to extracellular space, thereby decreasing the immediate post-transfusion haematocrit over the next 24 h (Audu et al. 2015). It is also possible that some erythrocyte destruction occurred and that alloantibodies were present but not detected by the cross-match reaction. Finally, the wildebeest underwent repeat surgery 24 h after the blood transfusion to replace screws in the plate on the tibia. Some blood loss did occur during this surgery and would have contributed to the decrease in haematocrit.

Interestingly, this wildebeest was hyperkalaemic and hypocalcaemic prior to blood transfusion. The most likely explanation for the hyperkalaemia is muscle damage associated with the fractures causing release of potassium from damaged cells into the systemic circulation (Perkins et al. 2007). This may have been perpetuated by manipulation of the limbs for radiographs and during surgical preparation. In humans, prolonged haemorrhagic shock has been shown to cause hyperkalaemia because of alteration in erythrocyte function (Illner, Cunningham & Shires 1982), and the acute blood loss that had occurred in this wildebeest may have played a role. A further contributing factor is the medetomidine used to immobilise the wildebeest. Alpha-2 adrenergic agonists inhibit insulin production through interaction at pancreatic *β* cells, thereby decreasing glucose uptake into cells (Rang et al. 2001). Potassium uptake by cells usually follows glucose uptake. Medetomidine has been associated with hyperkalaemia in domestic and wild canines and felines (Reilly et al. 2014). The hypocalcaemia was likely also associated with muscle trauma (Barstow 2017).

An area for improvement in the management of this particular case was the time taken to perform the transfusion. Ideally, the blood transfusion should have been started immediately after identification of the severe anaemia. However, it took 5 h to commence the transfusion. This time delay was partly because of the need to confirm the haematocrit using a microhaematocrit technique and the time taken to source a donor animal, after failure to identify an allodonor. Additional delays included the time to withdraw blood, the time to perform a cross-match analysis and the time to prepare a packed cell bag. Given the minimal chance of naïve reaction in ruminants, it would possibly have been better to start the transfusion without the cross-match results to increase oxygen delivery as quickly as possible.

## Conclusion

Xenotransfusion using blood from domestic ruminants represents a life-saving short-term emergency treatment of anaemic hypoxia in wild ungulates. Domestic goats could be used as blood donors for rare ungulates where allodonors are not available.
